# Bloodstream and catheter-related infections due to different clones of multidrug-resistant and biofilm producer *Corynebacterium striatum*

**DOI:** 10.1186/s12879-019-4294-7

**Published:** 2019-07-29

**Authors:** Juliana Nunes Ramos, Cassius Souza, Yuri Vieira Faria, Eliane Cristine da Silva, João Flávio Carneiro Veras, Paulo Victor Pereira Baio, Sérgio Henrique Seabra, Lilian de Oliveira Moreira, Raphael Hirata Júnior, Ana Luíza Mattos-Guaraldi, Verônica Viana Vieira

**Affiliations:** 1 Laboratório de Difteria e Corinebactérias de Importância Clínica, Faculdade de Ciências Médicas, Centro Colaborador e Referência para pesquisa de Difteria/Ministério da Saúde, Universidade do Estado do Rio de Janeiro (UERJ), Rio de Janeiro, Brazil; 2 Instituto Nacional de Controle de Qualidade em Saúde, Fundação Oswaldo Cruz, INCQS/FIOCRUZ, Rio de Janeiro, Brazil; 3 Laboratório Interdisciplinar de Pesquisas Médicas (LIPMED), Instituto Oswaldo Cruz, Fundação Oswaldo Cruz, Av. Brasil, 4365, Pavilhão Cardoso Fontes, 10 andar, sala 17, Manguinhos, Rio de Janeiro, 21040-900 Brazil; 4 Laboratório de Tecnologia em Bioquímica e Microscopia, Centro Universitário Estadual da Zona Oeste, Rio de Janeiro, Brazil; 5 Laboratório de Bacteriologia e Imunologia Clínica, Universidade Federal do Rio de Janeiro, Rio de Janeiro, Brazil

**Keywords:** Antimicrobial multiresistance, Bacteremia, Biofilm, Catheter-related infection, *C. striatum*, Nosocomial outbreak

## Abstract

**Background:**

*Corynebacterium striatum* is an emerging multidrug-resistant (MDR) pathogen associated with immunocompromised and chronically ill patients, as well as nosocomial outbreaks. In this study, we characterized 23 MDR *C. striatum* isolated of bloodstream and catheter-related infections from a hospital of Rio de Janeiro.

**Methods:**

*C. striatum* isolates were identified by 16S rRNA and *rpoB* genes sequencing. The dissemination of these isolates was accomplished by pulsed-field gel electrophoresis (PFGE). All isolates were submitted to antimicrobial susceptibility testing by disk diffusion and by minimum inhibitory concentration using E-test strips methods. Antimicrobial resistance genes were detected by polymerase chain reaction. Quantitative tests were performed on four different abiotic surfaces and the ability to produce biofilm on the surface of polyurethane and silicone catheter was also demonstrated by scanning electron microscopy.

**Results:**

Eleven PFGE profiles were found. The PFGE profile I was the most frequently observed among isolates. Five different MDR profiles were found and all PFGE profile I isolates presented susceptibility only to tetracycline, vancomycin, linezolid and daptomycin. Only the multidrug-susceptible isolate did not show mutations in the quinolone-resistance determinant region (QRDR) of the *gyrA* gene and was negative in the search of genes encoding antibiotic resistance. The other 22 isolates were positive to resistance genes to aminoglycoside, macrolides/lincosamides and chloramphenicol and showed mutations in the QRDR of the *gyrA* gene. Scanning electron microscopy illustrated the ability of MDR blood isolate partaker of the epidemic clone (PFGE profile I) to produce mature biofilm on the surface of polyurethane and silicone catheter.

**Conclusions:**

Genotyping analysis by PFGE revealed the permanence of the MDR PFGE profile I in the nosocomial environment. Other new PFGE profiles emerged as etiologic agents of invasive infections. However, the MDR PFGE profile I was also found predominant among patients with hematogenic infections. The high level of multidrug resistance associated with biofilm formation capacity observed in MDR *C. striatum* is a case of concern.

**Electronic supplementary material:**

The online version of this article (10.1186/s12879-019-4294-7) contains supplementary material, which is available to authorized users.

## Background

*Corynebacterium* genus consists of Gram-positive aerobic or anaerobic facultatively pleomorphic rods with a high G + C content DNA. Some species are part of human skin or mucosa [1, 2]. *Corynebacterium striatum* has been increasingly associated with severe infections in both immunocompetent and immunocompromised hosts [3, 4]. However, *C. striatum* isolates have been included among the etiologic agents of bacteremia with or without central venous catheter (CVC) in place [5, 6], endocarditis [7], breast abscesses [8], septic arthritis [2], osteomyelitis [4] and several other invasive diseases. In addition, studies have evidenced *C. striatum* as an emerging multidrug-resistant (MDR) pathogen related to nosocomial outbreaks in several countries [9–16].

The main risk factors for acquisition MDR *C. striatum* infections highlighted by Verroken et al. [13] were: prolonged hospital stay, advanced stage of chronic obstructive pulmonary disease, recent administration of antibiotics and exposure to an invasive diagnostic procedure. Besides, empirical antibiotic therapy may select MDR Gram-positive skin flora that can become the etiologic agent of nosocomial invasive diseases [17]. The emergence of MDR *C. striatum* and its involvement in nosocomial infections require appropriate interpretive criteria to the selection of the adequate antibiotic therapy [16].

Most reports of nosocomial infections and outbreaks caused by *C. striatum* mainly encompassed the respiratory tract [10, 13, 18]. On the other hand, few studies have investigated bloodstream and catheter-related infections by *C. striatum* [15, 19–21]. In a Brazilian tertiary care hospital located at Rio de Janeiro metropolitan area, a nosocomial outbreak caused by MDR *C. striatum* mostly isolated from tracheal aspirates samples was initially verified in 2009 [12]. Subsequently, cases of bloodstream and catheter-related infections caused by *C. striatum* isolates were noticed in the same hospital. In the present study, we aimed to investigate the clonal relationship, antimicrobial susceptibility profiles, ability of biofilm formation, molecular detection of resistance genes to amynoglycosides, quinolones, compounds of the MLSB group (macrolides and lincosamides) and chloramphenicol of these *C. striatum* invasive isolates.

## Methods

### Study design and origin of bacterial isolates

Twenty three *C. striatum* isolates recovered from blood and catheter samples of 21 hospitalised patients with signs and symptoms of bacteremia (*n* = 13) and catheter-related infections (*n* = 10) were analysed for microbiological features **(**Table 1**)**. Two of these isolates (2023 and 2038, PFGE profiles I and II, respectively) were previously studied by Baio and co-workers [12]. The patients were hospitalised during a 42-month period (January 2009 – February 2013) in 13 different wards of Hospital Universitário Pedro Ernesto (HUPE) - a tertiary care hospital belong to Universidade Estado Rio de Janeiro (UERJ) (*n* = 22) and Hospital Municipal Jesus (HMJ) (n = 1), both located at the metropolitan area of Rio de Janeiro, RJ, Brazil. The 2296 isolate came from a patient with CVC-related infection from HMJ and it was sent to the Laboratório de Difteria e Corinebactérias de Importância Clínica/UERJ for identification of the pathogen. *C. striatum* was isolated from blood samples and catheters segments of patients with signs and symptoms of bacterial infections as part of the medical care procedures of both hospitals. The consent to participate was not required because all the investigated isolates were taken as a part of standard care (diagnostic purposes). This study focused on bacteria and no identifiable human data were used. All the isolates were deposited in the CBAS/Fiocruz (Coleção de Bactérias do Ambiente e Saúde of Fundação Oswaldo Cruz) culture collection.

**Table 1:** Origin and PFGE profiles of 23 *Corynebacterium striatum* isolates from blood (*n* = 13) and catheter-related (*n* = 10) infections

Patient/ Isolation date	Isolates	Hospital wards	Gender	Isolation site	Culture	PFGE profiles ^a^
Aug2009	2023	General ICU^b^	M^c^	Blood	Pure	I
Sept2009	2038	Infectious diseases	F^d^	Blood	Pure	II
Apr2010	2089	General ICU	M	Catheter tip	Pure	I
Apr2010	2091	Infectious diseases	F	Blood	*+Acinetobacter* sp.	I
May2010	2103	Infectious diseases	F	Catheter tip	Pure	VIII
Aug2010	2130	Dermatology	F	Blood	Pure	V
Feb2011	2228	Cardiac ICU	F	Blood	Pure	VI
Feb2011;	2230;	Clinical ICU	M	Blood	Pure	VI
March2011	2237			Blood	Pure	VI
March2011	2243	General care	F	Blood	Pure	I
March2011	2245	General ICU	M	Catheter tip	Pure	I
June2011	2285	General ICU	F	Central venous catheter	Pure	I
July2011	2296	HMJ-RJ†	M	Central venous catheter	NI^e^	VII
Aug2011	2308	Hematology	M	Blood	Pure	I
Aug2011	2316	Orthopedic	M	Blood	+ CNS^g^	I
Oct2011	2324	Pulmonary	F	Catheter tip	+ CNS	Ia
Apr2012	2376	Neonatal ICU	F	Catheter tip	Pure	X
May2012	2390	General care	F	Catheter tip	Pure	VIa
June2012	2401	Thoracic Surgery	M	Blood	Pure	II
Aug2012;	2425;	Hematology	F	Blood	Pure	IX
Sept2012	2432	Pediatric		Blood	Pure	IX
Nov2012	2439	General care	M	Catheter tip	Pure	Ib
Feb2013	2454	General care	F	Catheter tip	+ CNS/ *KlebsiellaPneumoniae*	I

^a^ I- X, PFGE profiles. Previously described non-MDR isolates of PFGE types III and IV were not found in patients with blood and catheter-related infections (Baio et al. 2013); ^b^ ICU, intensive care unit; ^c^ M, male; ^d^F, female; ^e^ NI, Not informed; ^f^HMJ – RJ, Hospital Municipal Jesus - Rio de Janeiro; ^g^CNS, Coagulase-negative staphylococci

### Bacterial isolates collection, culture conditions and phenotypic identification procedures

Clinical specimens were inoculated in Bactec Plus anaerobic/aerobic vials and analysed in a Bactec 9240 continuous-monitoring system (Becton-Dickinson Microbiology System, Cockeysville, MD, USA). Corynebacterium-like colonies were selected for identification when they were grown in significant numbers (> 15 colonies) or in pure culture from blood or catheter samples, as recommended by Maki’s semi quantitative method to distinguish infection (> 15 colonies) from contamination of catheter-tips [22]. Bacterial isolates were identified by conventional phenotypic characterization [12, 23] and Vitek 2 (bioMérieux, France) using Anaerobe and Corynebacterium card.

### *C. striatum* molecular identification by 16S rRNA and *rpoB* genes sequencing

Molecular identification was performed according with protocols previously described [12]. The 16S rRNA and rpoB genes sequences were compared to type strains sequences available in the National Center for Biotechnology Information (ncbi.nlm.nih.gov) using the BLAST algorithm and/or the Eztaxon server [24].

### Pulsed-field gel electrophoresis (PFGE)

Pulsed-field gel electrophoresis was performed as described by Baio and co-workers [12]. PFGE banding profiles were analyzed using visual comparison among the isolates, according to the criteria established by Tenover and co-workers [25] and by automated analysis using the BioNumerics Fingerprinting software (Version 4.0, Applied Math, Belgium). PFGE profiles were identified by roman numerals and subtypes were identified by roman numerals followed by a letter. The similarity index of the isolates was calculated using the Dice correlation coefficient with a band position tolerance of 1%. The unweighted-pair group method using average linkages (UPGMA) was used to construct a dendrogram. PFGE profiles that showed similarity coefficient ≥ 85% were considered genetically related [26, 27]. The isolates previously studied, 1961 (PFGE profile III) and 1954 (PFGE profile IV) were used as controls [12].

### Antimicrobial susceptibility testing

Antimicrobial susceptibility profiles were determined by the disk diffusion and by minimum inhibitory concentration (MIC) using E-test strips methods in cation-adjusted Mueller-Hinton agar supplemented with 5% sheep blood using inoculum equivalent to a 0.5 McFarland standard. Seven antibiotic disks (Oxoid, Hampshire, United Kingdom) were used: clindamycin (2 μg), moxifloxacin (5 μg), gentamicin (10 μg), rifampicin (5 μg) and vancomycin (5 μg), according Brazilian Committee on Antimicrobial Susceptibility Testing – BrCAST [28]. Another three antimicrobials imipenem (10 μg), erythromycin (15 μg) and chloramphenicol (30 μg) were interpreted in accordance to criteria defined by BrCAST for *Staphylococcus* spp. These antibiotics are not considered in Clinical and Laboratory Standards Institute (CLSI) and BrCAST/EUCAST guidelines for *Corynebacterium* spp. Linezolid 30 μg was interpreted in accordance to criteria defined by CLSI [29] for *Staphylococcus* spp. The BrCAST document recommends the use of linezolid 10 μg for antimicrobial susceptibility testing, but this is not found commercially available in Brazil.

Antimicrobial susceptibility to daptomycin, penicillin, ciprofloxacin and tetracycline were determined by MIC using E-test strips (AB Biodisk, Sweden). Interpretation of values to penicillin, ciprofloxacin and tetracycline were performed also according BrCAST [28]. Due to absence of daptomycin breakpoints in this guideline, the interpretation of values was performed according to CLSI M45-A2 document [30]. MDR was defined as acquired non-susceptibility to at least one agent in three or more antimicrobial categories [31].

### Amplification and sequencing of the genes related to resistance

The amplification and sequencing of the QRDR region of the *gyrA* gene was performed following protocols described by Sierra and co-workers [32]. Mutations in the QRDR region of the *gyrA* gene were identified by aligning of amino acids sequences and compared to the sequence of *C. striatum* ATCC 6940 (GenBank accession number AY559038) using Clustal X program [33]. The 23 *C. striatum* isolates also were screened for the presence of genes codifying antibiotic resistance, *ermX* (macrolides and lincosamides), *aphA* (amynoglycoside) and *cmx* (chloramphenicol), by PCR using primers specific for each gene [11, 34]. For each gene, one amplicon was purified and sequenced according with protocols previously described by Baio and co-workers [12].

### Semi-quantitative analyses of biofilm formation on polyurethane and silicone catheter surfaces

Sterile 4 cm segments of 16-gauge percutaneous nephrostomy polyurethane and silicone catheters were immersed in TSB containing 10^6^ CFUml^− 1^ of *C. striatum* and incubated at 37 °C for 48 h then Maki’s semi-quantitative roll plate technique were performed. Basically, after washing (three times) with phosphate buffered saline (PBS) 0.1 M pH 7.2, contaminated abiotic substrates were rolled up on Columbia agar plates supplemented with 5% sheep blood (Oxoid, Germany) for 48 h at 37 °C were analyzed presence of bacterial carpet [23, 35].

### Quantitative tests of biofilm formation on different on catheter abiotic surfaces

Quantitative analysis of viable sessile cells of representative isolates of PFGE profile I (2023) and II (2038) was evaluated by quantitative tests based on previously described methods [35] and using the following abiotic substrates: surfaces of glass tubes, Thermanox cover slips (Nunc), catheters fragments of polyurethane and metal tips (Intracath; Deseret Pharmaceutical Co., Sandy, Utah). Each experiment was carried out in triplicate and repeated three times. Results of the viable cell counts of experiments performed with the 2023 and 2038 isolates were compared using one-way analysis of variance (ANOVA) and Tukey’s multiple-comparison post-test. The values of *p* < 0.05 were considered statistically significant. Statistical analyses were performed using GraphPad Prism version 6 (GraphPad Software, San Diego, CA).

### Morphological aspects of biofilm formation polyurethane and silicone catheters

The biofilm produced (48 h incubation) on the surface of in vitro prepared on fragments of polyurethane and silicone catheters by *C. striatum* isolates from blood, 2023 (PFGE profile I) and 2038 (PFGE profile II), were demonstrated by Scanning Electron Microscopy (SEM). Specimen preparation and staining protocol for SEM were performed as described by Souza and co-workers [35]. Catheter segments infected in vitro with *C. striatum* 1987 (PFGE profile I), 2369 (PFGE profile II), 1961 (PFGE profile III) and 1954 (PFGE profile IV) isolates were used as positive controls [35].

## Results

### *C. striatum* genotypic identification

The analysis of 16S rRNA and *rpoB* genes sequences from 23 isolates from blood and catheter samples exhibited the highest similarity values with *C. striatum* ATCC 6940 type strain, ranging from 99.77–99.12% and 99.28–98.65%, respectively. GenBank accessions for *C. striatum* 16S rRNA and *rpoB* genes were deposited: KJ934779 to KJ934789, KM001910 to KM001914, KJ855309 to KJ855313, KR010627 to KR010645, KR020513 and KR020514.

### PFGE analysis

**Fig. 1:**
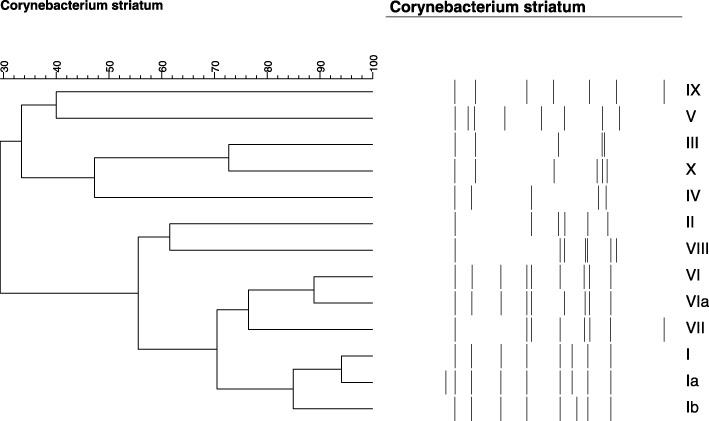
Dendrogram generated by Dice/UPGMA analysis (Bionumerics, Applied Maths) of *Swa*I PFGE profiles of *C. striatum* isolated from bloodstream infection. I- X, PFGE profiles. Previously described non-MDR isolates of PFGE types III and IV were not found in patients with blood and catheter-related infections [12]

Eleven PFGE profiles among *C. striatum* isolates were designated I, Ia, Ib, II, V, VI, VIa, VII, VIII, IX and X (Table 1; Fig. 1; Additional files 1 and 2). The PFGE profile I was the most frequently (*n* = 9) observed among isolates (Table 1). The PFGE profiles I, Ia and Ib showed similarity coefficient ≥ 85% were considered genetically related. The PFGE profiles VI and VIa showed similarity coefficient ≥ 90% were also considered genetically related. PFGE analysis indicated that bloodstream infections in cases 8 and 19 were due to particular clones of *C. striatum*: PFGE profiles VI (2230 and 2237 isolates) and IX (2425 and 2432 isolates), respectively. PFGE profile X was related to a case of catheter-related infection in a 3 months-old patient from Neonatal ICU. *C. striatum* PFGE profile VII was isolated only from patient with CVC infection from HMJ-RJ (Table 1). *C. striatum* PFGE profile I isolates were detected in patients with bloodstream and/or catheter-related infections during the years of 2009 (*n* = 1), 2010 (*n* = 2), 2011 (*n* = 5), 2013 (n = 1) but not 2012.

### Antimicrobial multiresistance profiles

Antimicrobial susceptibility profiles of 23 *C. striatum* isolates are displayed in Table 2. Except the 2376 isolate from Neonatal ICU, all *C. striatum* isolates, independent of their PFGE profiles and hospital settings, showed non-susceptibility to at least one agent in three or more antimicrobial categories and were consequently identified as MDR pathogens. Twenty-two *C. striatum* isolates exhibited five different multiresistance levels corresponding to five different MDR profiles: A, with resistance to 9 antimicrobial agents; B and B1, resistance to 8 antimicrobial agents; C, resistance to 7 antimicrobial agents; and D, with resistance to 6 antimicrobial agents. All *C. striatum* isolates related to bloodstream and catheter-related infections, representative of different PFGE profiles, showed susceptibility to tetracycline, vancomycin, linezolid and daptomycin. *C. striatum* isolates, independent of PFGE profiles showed resistance to penicillin (100%), ciprofloxacin and moxifloxacin (95.6%), clindamycin (95.6%), erythromycin (95.6%), gentamicin (91.3%), rifampin (52.2%), imipenem (76.2%) and chloramphenicol (95.6%). MDR profile A was demonstrated for 8 *C. striatum* PFGE profile I isolates. Moreover, MDR profile A was also shown for *C. striatum* isolates PFGE profiles Ib, II and VIII.

**Table 2:** Antimicrobial susceptibility profiles of 23 *Corynebacterium striatum* isolates from blood (*n* = 13) and catheter-related (*n* = 10) infections

Isolates	PFGE profiles	Penicillin	Ciprofloxacin	Moxifloxacin	Gentamicin	Clindamycin	Erythromycin	Rifampin	Imipenem	Chloramphenicol	Resistance Profile
2023	I	R	R	R	R	R	R	R	R	R	A
2089	I	R	R	R	R	R	R	R	R	R	A
2091	I	R	R	R	R	R	R	R	R	R	A
2245	I	R	R	R	R	R	R	R	R	R	A
2285	I	R	R	R	R	R	R	R	R	R	A
2308	I	R	R	R	R	R	R	R	R	R	A
2316	I	R	R	R	R	R	R	R	R	R	A
2454	I	R	R	R	R	R	R	R	R	R	A
2439	Ib	R	R	R	R	R	R	R	R	R	A
2038	II	R	R	R	R	R	R	R	R	R	A
2103	VIII	R	R	R	R	R	R	R	R	R	A
2243	I	R	R	R	R	R	R	S	R	R	B
2324	Ia	R	R	R	R	R	R	S	R	R	B
2228	VI	R	R	R	R	R	R	S	R	R	B
2230	VI	R	R	R	R	R	R	S	R	R	B
2237	VI	R	R	R	R	R	R	S	R	R	B
2390	VIa	R	R	R	R	R	R	R	S	R	B1
2401	II	R	R	R	R	R	R	S	S	R	C
2425	IX	R	R	R	R	R	R	S	S	R	C
2432	IX	R	R	R	R	R	R	S	S	R	C
2296	VII	R	R	R	R	R	R	S	S	R	C
2130	V	R	R	R	S	R	R	S	S	R	D
2376	X	R	S	S	S	S	S	S	S	S	E
Resistance %		100	95.6	95.6	91.3	95.6	95.6	52.2	76.2	95.6	

*R* resistant, *S* susceptible

### Molecular detection of resistance genes

The sequences of the QRDR region of the *gyrA* gene of 22 isolates characterized as resistant to fluoroquinolones were compared to that of the quinolone-susceptible *C. striatum* ATCC 6940 (MIC = 0.094 mg/L) and with our multidrug-susceptible (MDS) 2376 isolate (MIC = 0.125 mg/L). The relationships between the mutations in the QRDR region *gyrA* gene and the MICs of ciprofloxacin of the 23 isolates and *C. striatum* ATCC 6940 are summarized in Table 3. Twenty-one isolates showed MIC > 32 mg/L to ciprofloxacin, fifteen of them carried a mutation only the position 87 generating a change from Ser-87 to Val. The 2324 and 2401 isolates carried a double mutation, generating a change from Ser-87 to Val and Asp-91 to Asn. The 2130 isolate had MIC = 2 mg/L and carried a mutation only in position 87 (Ser-87 to Tyr). All sequences of QRDRs region *gyrA* gene were deposited in GenBank/NCBI under numbers MG010347-MG010369.

**Table 3:** Relationship between mutations in the QRDR region and the MICs for 23 *C. striatum* isolates and ATCC 6940

Number of isolates	Ciprofloxacin		GyrA (amino acids)	
	MIC (mg/L)	Phenotype	Position 87	Position 91
15	> 32	R	Val	Asp
2	> 32	R	Val	Asn
1	> 32	R	Phe	Asp
3	> 32	R	Phe	Ala
1	2	R	Tyr	Asp
1	0.125	S	Ser	Asp
ATCC 6940^a^	0.094	S	Ser	Asp

^a^ GenBank accession number AY559038

The genes codifying for antibiotic resistance, *ermX* (macrolides and lincosamides), *aphA* (amynoglycoside) and *cmx* (chloramphenicol) were found except in MDS 2376 isolate. Twenty-two isolates resistant to erythromycin, clindamycin and chloramphenicol presented *ermX* and *cmx* genes. These isolates were also *aphA*-positive and resistant to gentamicin, except for one isolate. The 2130 isolate was *aphA*-positive and susceptible to gentamicin, confirmed by MIC using E-test strip gentamicin. Analysis of genes sequences exhibited high similarity values (above 99%) with those deposited in NCBI. The sequences were deposited in GenBank/NCBI under numbers MF872250 to MF872252.

### Quantitative tests of biofilm formation on different catheter abiotic surfaces

**Fig. 2:**
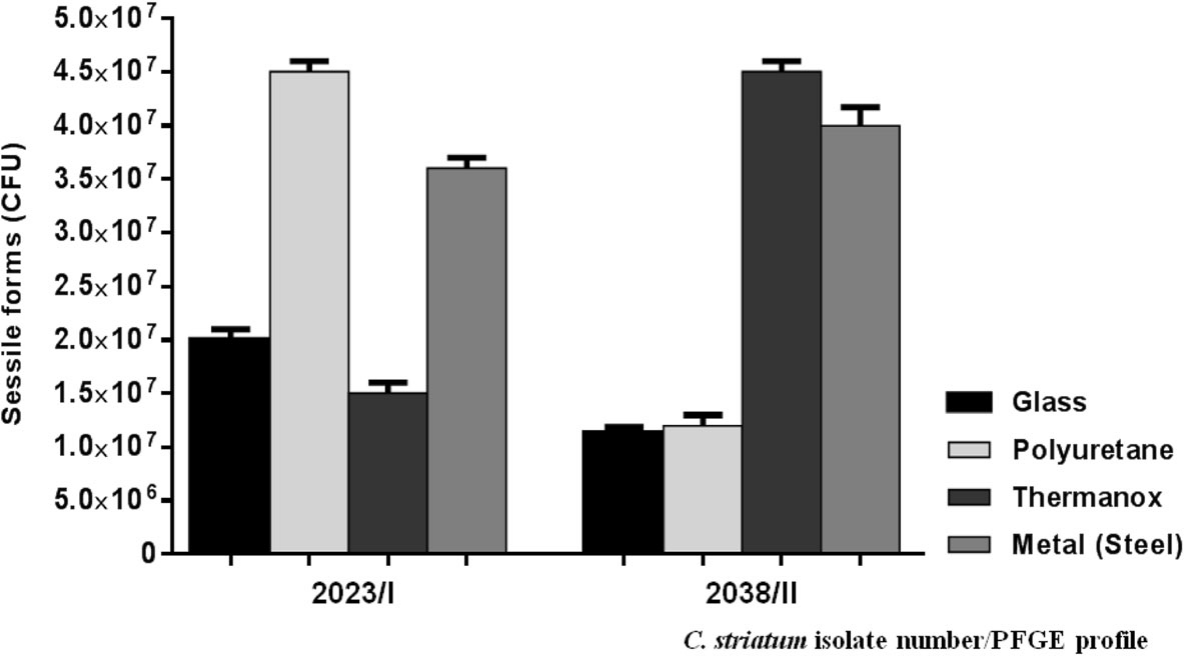
Adhesive levels, biofilm formation and survival on different types of abiotic surfaces of *C. striatum* (2023/PFGE profile I MDR and 2038/PFGE profile II MDR isolates) isolated from patients with bloodstream infection evaluated by quantitative tests: glass and polyurethane (hydrophilic and positively charged), polystyrene and thermanox (hydrophobic and negatively charged), and metal (catheter’s tips) surfaces. Mean values and standard deviations of three independent experiments and magnifications are shown in the figure

Results of quantitative analysis of viable sessile cells of MDR *C. striatum* isolates 2023 and 2038 (PFGE profiles I and II, respectively) are shown in Fig. 2**.** Viable sessile bacterial cells were detected 48 h post-infection on surfaces of all types of abiotic substrates tested, but at different levels. *C. striatum* 2023 isolate (PFGE profile I) showed a higher ability to adhere to glass surface, a hydrophilic and positively charged abiotic surface (*p* < 0.001). In addition to glass, *C. striatum* 2023 isolate also exhibited a significantly (p < 0.001) greater level of adherence to an abiotic hydrophilic (polyurethane) positively charged surface compared with *C. striatum* 2038 isolate (PFGE profile II). Conversely, *C. striatum* 2038 isolate exhibited a significantly (p < 0.001) greater level of adherence to negatively charged and hydrophobic polystyrene (Thermanox) surface compared with 2023/PFGE profile I isolate. Interestingly, both *C. striatum* isolates (PFGE profiles I and II) were able to strongly adhere and survive on the metal surface with similar intensities (*p* = 0.374) (Fig. 2).

### Semi-quantitative analyses biofilm produced in vitro model of catheter infection

**Fig. 3:**
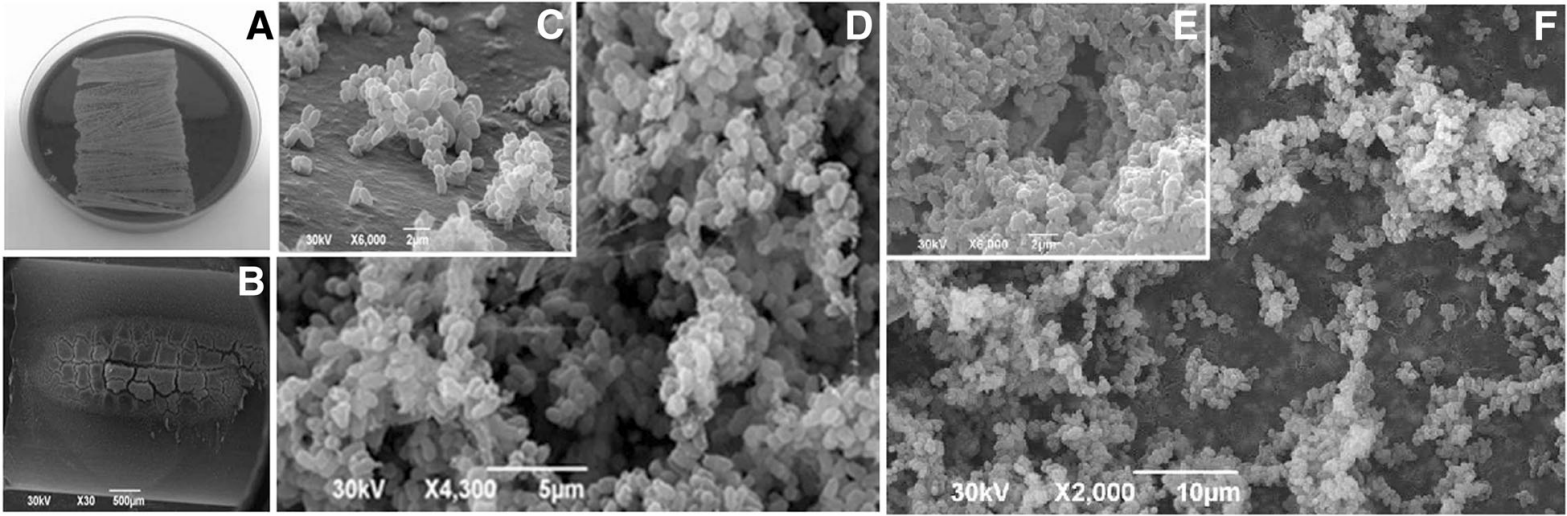
Biofilm formation (48 h incubation) on the surface of in vitro infected polyurethane and silicone catheters by the two *C. striatum* isolates from bloodstream infections: (**a-d**) 2023/PFGE profile I isolate; (**e, f**) 2038/PFGE profile II isolate. (**c**) Microcolony formation (a hallmark of biofilm formation) by autoaggregative *C. striatum* on catheter surface. SEM assays of biofilm formation on (**c, d)** polyurethane and (**e, f**) silicone catheters surfaces. (**d, e**) Presence of hollow voids indicative of mature biofilm formation

Results of the semi-quantitative roll plate method (> 15 CFU) showed extensively adherent viable sessile forms for both *C. striatum* isolates (2023 and 2038) on fragments of polyurethane and silicone catheters, as illustrated in Fig. 3a.

### Morphological aspects of biofilm formation on polyurethane and silicone catheters evaluated by SEM

Micrographs illustrating biofilm formation on the surface of polyurethane and silicone catheters by *C. striatum* 2023/PFGE profile I (Fig. 3b-d) and 2038/PFGE profile II (Fig. 3e, f) isolates demonstrated by SEM are displayed in Fig. 3b-f.; Fig. 3c showed microcolony formation (a hallmark of biofilm formation) by auto aggregative *C. striatum* on polyurethane surface. SEM assays also evidenced the presence of hollow voids indicative of mature biofilm formation on surfaces of polyurethane (Fig. 3d, e) and silicone (Fig. 3f) catheters.

## Discussion

Antimicrobial resistance has a major impact on human health [36]. At present, reports on the emergence and spread of multiresistant bacterial species are important to support the progress of resistance control policies. Our data show bloodstream and catheter-related infections caused by different clones of MDR *C. striatum* in Brazil. In accordance to Chen and co-workers [37], our findings emphasize that *C. striatum* from blood and catheter segments should not be considered only as contaminant, since in our study most of the isolates were found in pure cultures (82%) or in significant numbers.

In Brazil the isolation of *C. striatum* from hospitalized patients with signs and symptoms of infection was observed in some studies [38–40]. In a previous study, we documented four PFGE profiles during a nosocomial outbreak caused by *C. striatum* in Rio de Janeiro, Brazil. MDR clones related to the profiles of PFGE I and II were predominant. In that opportunity only two isolates of PFGE I and II profiles were isolated from blood samples [12]. Due to subsequent increased number of cases of bloodstream and catheter-related infections caused by *C. striatum* isolates in HUPE, current investigation of the clonal relationship of these *C. striatum* isolates revealed the permanence of the MDR PFGE profiles I and II in the nosocomial environment as invasive clones. However, the PFGE I profile was found predominant among patients with hematogenic infections. In addition, other new MDR PFGE profiles (V to IX) emerged as etiologic agents of bloodstream and catheter-related infections. Interestingly, one non-multiresistant clone (PFGE profile X) was also related to a case of catheter-related infection in the newborn.

PFGE is a valuable tool to investigate the clonal relatedness of microbial strains during nosocomial outbreaks. Several nosocomial infections and outbreaks studies have employed this methodology for typing of *C. striatum* isolates [13, 15, 16, 18]. PFGE is a stable and reproducible genotyping method, however, it is time consuming and standardizations for inter-laboratory comparisons do not exist for *C. striatum* isolates genotyping. Thus, the method is only applicable to compare isolates for regional epidemiology surveillance. Gomila and co-workers described the development of a multilocus sequence typing (MLST) scheme for *C. striatum*. [9]. However, the proposed MLST scheme has not been adopted by scientific community, perhaps due to the limited number of genes (ITS1 region, *gyrA* and *rpoB*) that comprise it. PFGE has limitations but it is the tool available so far for the discrimination of this bacterial species. Future researches are required to evaluate genotyping methods that provide useful data for global surveillance of infections caused by *C. striatum*.

Antimicrobial susceptibility testing remains rarely performed on *Corynebacterium* spp. in many laboratories [41]. The method of susceptibility by disk-diffusion is widely used by microbiology laboratories in Brazil and in other countries [12, 42–44]. Moreover, CLSI guidelines do not provide breakpoints for disk-diffusion while BrCAST, based in EUCAST document, provides breakpoints for corynebacteria susceptibility testing only for some antibiotics [28, 29], excluding various antimicrobials, such as cephalosporins, carbapenems and lipopeptides, thus many researchers often use staphylococcal breakpoints [12, 16, 45]. Susceptibility testing should be performed on clinically significant *C. striatum* isolates. However, studies suggest that according to the severity of the infection, empiric treatment should be carried out with vancomycin and linezolid because of low levels of susceptibility to other antimicrobials [13, 46]. In some studies, therapy with an association of at least two of the following antimicrobial agents has been reported: vancomycin, rifampin, linezolid and daptomycin [47, 48].

Resistance to imipenem by *C. striatum* isolates has been related in some countries, such as Japan [49], Spain [9, 10] and Italy [11]. Most of our *C. striatum* isolates showed resistance to imipenem (76,2%). Combination therapy that includes imipenem for the treatment of MDR *C. striatum* infections in Brazil should be more prudent.

Effective treatment of corynebacterial infections with daptomycin has been reported in the literature [48, 50]. In this study, all *C. striatum* isolates showed susceptibility to daptomycin. However, resistance to daptomycin in *C. striatum* has been documented and may occur during therapy in patients with invasive infections. Consequently, some authors recommend caution in daptomycin monotherapy for treatment of these infections [41, 51].

Some mechanisms of antimicrobial resistance have been reported in *Corynebacterium* species. Studies of the sequences encoding the A subunit of the gyrase enzyme in strains of *C. striatum, Corynebacterium amycolatum* and *Corynebacterium macginley* have shown that resistance to fluoroquinolones is associated with mutations of a spontaneous nature in this gene and depends on the number of mutations and the type of amino acid that was exchanged [16, 32, 52, 53]. Twenty-two isolates were resistant to the quinolones tested. The combinations of amino acids Val/Asn and Tyr/Asp in positions 87 and 91 of QRDR region *gyrA* gene, respectively, found in three of our isolates have not been described in the literature for *Corynebacterium* species until the present moment, conferring resistance to ciprofloxacin and moxifloxacin.

The *ermX* gene (*erythromycin ribosome methylation*) encoding the rRNA methylase enzyme leads to simultaneous resistance to macrolides, lincosamides and streptogramins B (MLSB) [45, 54]. This gene was found on chromosomes, plasmids and transposons of corynebacteria. We found this gene in 22 isolates, except in the MDS 2376 isolate, which may indicate that the *ermX* gene can be involved in the clindamycin and erythromycin resistance phenotype of our isolates.

Antimicrobial susceptibility studies have shown *C. striatum* isolates resistant to aminoglycosides. Consequently, the use of aminoglycosides as second-line complementary antimicrobial for treatment of *C. striatum* infections should be cautious [55, 56]. The mechanisms of aminoglycoside resistance most common are the aminoglycoside modifying enzymes (AME’s) classified in 3 classes: *aac* (*acyl-coenzyme A-dependent acetyltransferase*), *ant* (*nucleoside triphosphate-dependent nucleotidyl transferases*) and *aph* (*nucleoside triphosphate-dependent phosphotransferases*). These enzymes are often disseminated by various mobile genetic elements and many aminoglycosides can be inactivated by more than one enzyme [57]. In this study, the *aph* gene was found in 22 isolates, except in the MDS 2376 isolate, but one *aph*-positive isolate showed susceptibility to gentamicin, confirmed by MIC E-test strip (MIC = 0.12 mg/L, according to BrCAST). Future analyzes of the region where the gene is located and other mechanisms of resistance to aminoglycosides in all *aph*-positive isolates should be made.

The *cmx* gene is responsible for coding the efflux protein to chloramphenicol and has already been found in transposons, plasmids and genomes of *Corynebacterium* species [58–60]. All isolates *cmx*-positive were resistant to chloramphenicol. The *cmx* gene sequence showed similarity above 99% with the sequences of *cmx* gene found in chromosomes, plasmid and genomic island of *Pseudomonas aeruginosa* and other *Corynebacterium* species deposited in GenBank/NCBI.

Biofilm is a structure that facilitates several bacterial processes influencing virulence and resistance to antimicrobials such as adhesion capacity, metabolite exchange, cellular communication, protection to antimicrobials, protection against host immune attacks. Consequently, the formation of bacterial biofilms leads to an increase in healthcare costs and extend hospitalization [61, 62]. Previously, Souza and co-workers verified that *C. striatum* PFGE profiles I to IV formed biofilm on hydrophilic and hydrophobic surfaces. *C. striatum* PFGE profile I, predominant isolated from nosocomial outbreak, showed the greatest ability to adhere to all surfaces produced much more biofilm than the other profiles [35]. In Japan, Qin and co-workers observed that all 6 *C. striatum* isolates identified as predominant PFGE profile had high ability to produce biofilm in glass cover-slips after 72 h post-incubation [15]. In the present study, biofilm formation and survival on four abiotic surfaces (glass, metal, polyurethane and silicone) were demonstrated 48 h post-incubation of bacterial cells representative of MDR *C. striatum* PFGE profiles I and II isolated from patients with bloodstream infections. Similar to *C. striatum* PFGE profile I isolated from patients undergoing endotracheal intubation procedures, PFGE profile I isolated from bloodstream and catheter-related infections also showed a higher ability to adhere to and to survive on abiotic surfaces of medical devices including those used in invasive procedures. MDR *C. striatum* viable cells were able to multiply and to produce mature biofilms on both types of catheter surfaces.

## Conclusions

The ability of some *C. striatum* clones to produce biofilm on different types of abiotic surfaces may contribute to the pathogenicity favoring bacterial invasive potential and establishment of bloodstream and catheter-related infections, in addition to permanence in hospital environment and dissemination of antimicrobial resistance. The potential of *C. striatum* to cause infection should not be underestimated. Therefore, antimicrobial susceptibility testing should be performed on clinically significant *C. striatum* isolates. Medical surveillance programs should include control strategies in order to decrease potential risk factors of nosocomial infections and outbreaks due to *C. striatum.*

## Additional files

Additional file 1: **Figure S1.** Pulsed-field gel electrophoresis (PFGE) profiles of Brazilian *Corynebacterium striatum* isolates from blood and catheter segments. Lane 1: λ DNA ladder PFGE marker; lane 2: profile VIII (isolate 2103); lanes 3, 5 and 6: profile I (isolates 2316, 2439 and 2023, respectively); lane 4: profile Ia (isolate 2324); lane 7: profile X (isolate 2376); lane 8: profile VIa (isolate 2390); lane 9: profile IX (isolate 2425) and lane 10: profile Ib (isolate 2454). (TIF 523 kb)

Additional file 2: **Figure S2.** Pulsed-field gel electrophoresis (PFGE) profiles of Brazilian *Corynebacterium striatum* isolates from blood and catheter segments. Lane 1: λ DNA ladder PFGE marker; lanes 2, 3 and 7: profile I (isolates 2089, 2091 and 2023, respectively); lane 4: profile V (isolate 2130); lanes 5, 6 and 9: profile VI (isolates 2228, 2230 and 2237, respectively); lane 8: profile VII (isolate 2296) and lane 10: profile IV (isolate 1954 – control). (TIF 469 kb)

## Data Availability

All data generated of analyses during this study are included in this published article and its additional files. The datasets including sequencing data used and analyzed during the current study are deposited in the Genbank. GenBank accessions for *C. striatum* 16S rRNA and *rpoB* genes were deposited: KJ934779 to KJ934789, KM001910 to KM001914, KJ855309 to KJ855313, KR010627 to KR010645, KR020513 and KR020514. *C. striatum* ATCC 6940 (GenBank accession number AY559038)All sequences of QRDRs region *gyrA* gene were deposited in GenBank/NCBI under numbers MG010347-MG010369.

## Abbreviations

CFU: Colony-forming unit
CLSI: Clinical da Laboratory Standards Institute
CVC: Central venous catheter
HMJ: Hospital Municipal de Jesus
HUPE: Hospital Universitário Pedro Ernesto
ICU: Intensive care unit
MDR: Multidrug-resistant
MDS: Multidrug-susceptible
MIC: Minimum inhibitory concentration
MLSB: Macrolides, lincosamides and streptogramins B
PFGE: Pulsed-field gel electrophoresis
QRDR: Quinolone-resistance determinant region
UERJ: Universidade do Estado do Rio de Janeiro
UPGMA: Unweighted-pair group method using average linkages
