# Supplementary figures and images for: Bloodstream and catheter-related infections due to different clones of multidrug-resistant and biofilm producer Corynebacterium striatum
Source: BMC Infect Dis. 2019 Jul 29;19:672. doi: 10.1186/s12879-019-4294-7 (PMC6664767; doi:10.1186/s12879-019-4294-7)

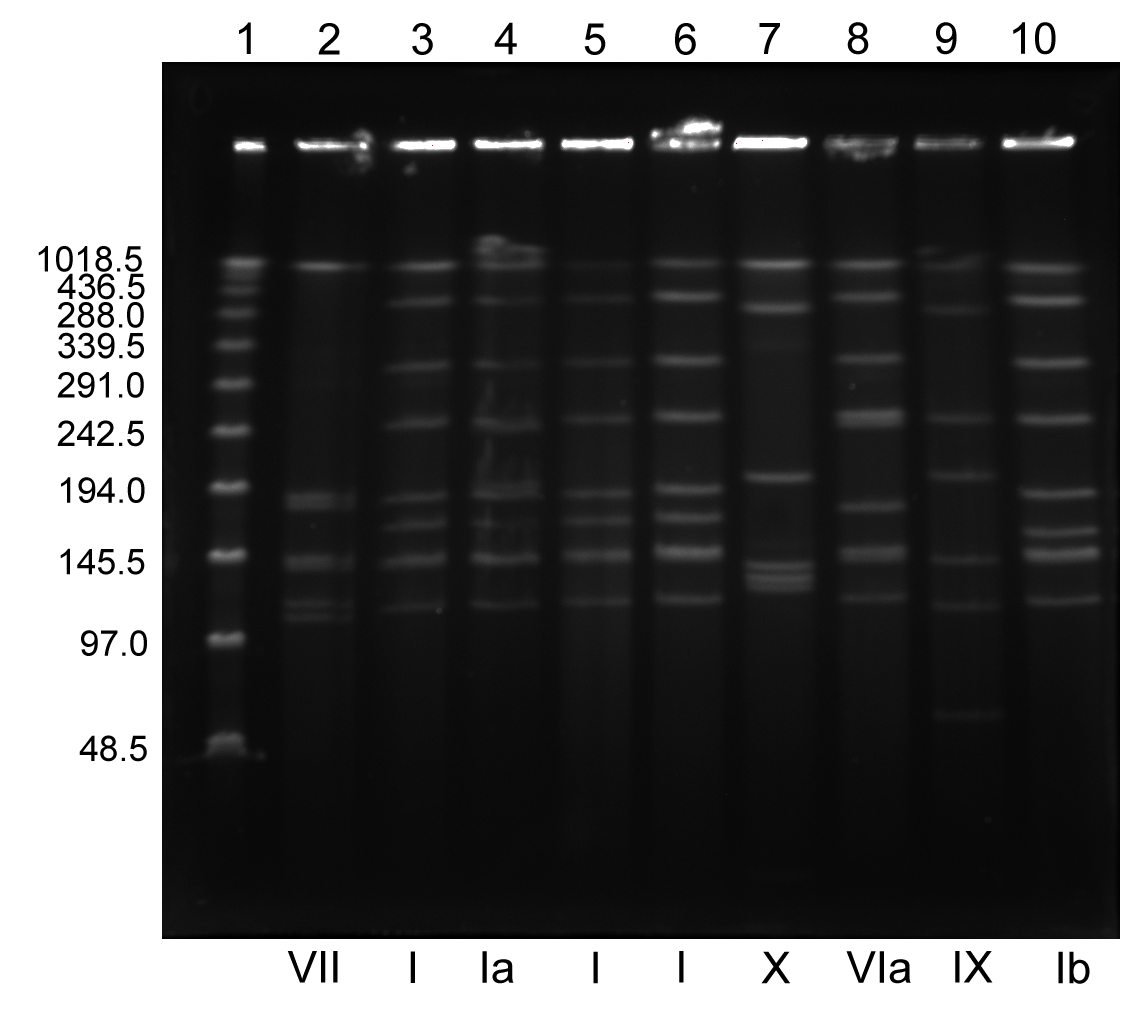

Supplement: Supplementary file 1 — Figure S1. Pulsed-field gel electrophoresis (PFGE) profiles of Brazilian Corynebacterium striatum isolates from blood and catheter segments. Lane 1: λ DNA ladder PFGE marker; lane 2: profile VIII (isolate 2103); lanes 3, 5 and 6: profile I (isolates 2316, 2439 and 2023, respectively); lane 4: profile Ia (isolate 2324); lane 7: profile X (isolate 2376); lane 8: profile VIa (isolate 2390); lane 9: profile IX (isolate 2425) and lane 10: profile Ib (isolate 2454). (TIF 523 kb) [file 12879_2019_4294_MOESM1_ESM.tif]

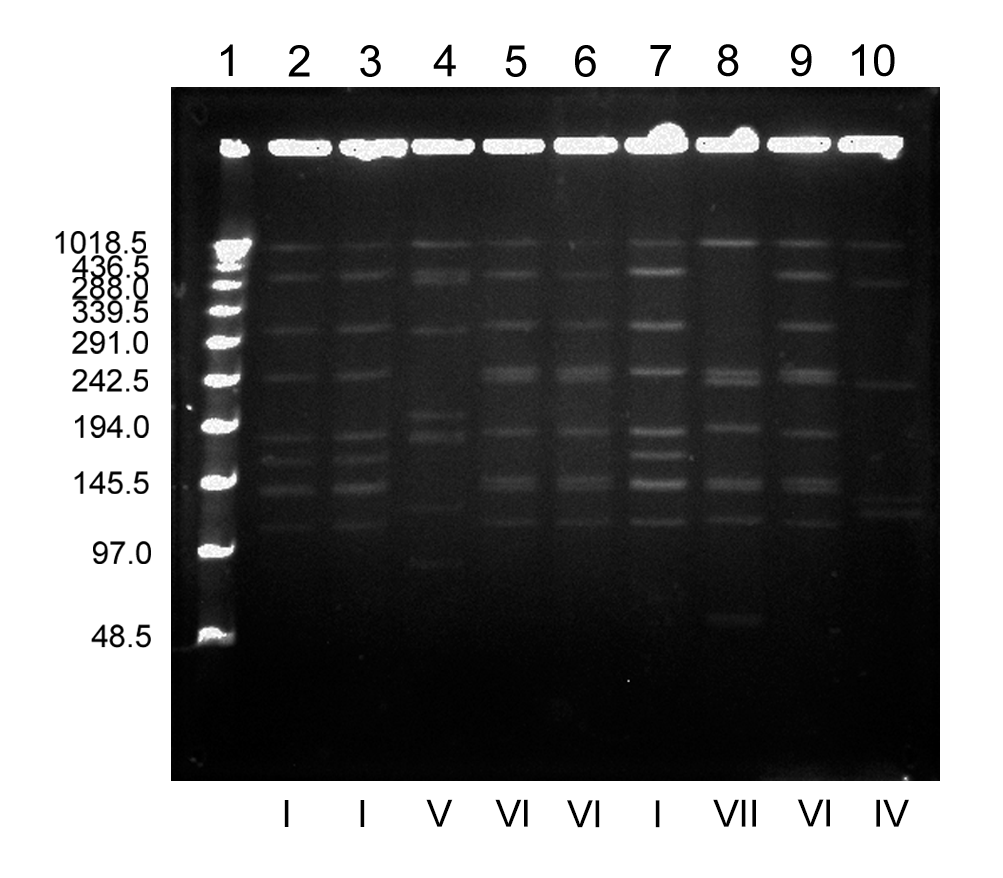

Supplement: Supplementary file 2 — Figure S2. Pulsed-field gel electrophoresis (PFGE) profiles of Brazilian Corynebacterium striatum isolates from blood and catheter segments. Lane 1: λ DNA ladder PFGE marker; lanes 2, 3 and 7: profile I (isolates 2089, 2091 and 2023, respectively); lane 4: profile V (isolate 2130); lanes 5, 6 and 9: profile VI (isolates 2228, 2230 and 2237, respectively); lane 8: profile VII (isolate 2296) and lane 10: profile IV (isolate 1954 – control). (TIF 469 kb) [file 12879_2019_4294_MOESM2_ESM.tif]
